# Relationship between the efficacy of micro-implant-assisted rapid maxillary arch expansion and maturation of the midpalatal sutures

**DOI:** 10.2340/aos.v85.45902

**Published:** 2026-05-04

**Authors:** Suna Li, Jincong Tian, Yuanyuan Li, Wenshang Song

**Affiliations:** a Department of Orthodontics, Hengshui People’s Hospital, Hengshui, Hebei, China; b Department of Pediatric Dentistry, Tianjin Stomatological Hospital, School of Medicine, Nankai University, China

**Keywords:** Midpalatal suture, mini-screw, rapid maxillary expansion, cone beam computed tomography

## Abstract

**Objective:**

To investigate the relationship between the efficacy of micro-implant-assisted rapid palatal expansion (MARPE) and the degree of midpalatal suture (MBT) fusion.

**Methods:**

Forty patients with maxillary transverse hypoplasia from Hengshui People’s Hospital from January 2022 to August 2024 were recruited for MARPE treatment. These patients were divided into unfused (stages B and C, *n* = 18), initiation fusion (stage D, *n* = 10), and complete fusion groups (stage E, *n* = 12) according to the degree of MBT fusion based on the staging method for palatal sutures. Cone beam computed tomography (CT) and gypsum models were created before and on the day after arch expansion. The differences in maxilla, alveolar bone, tooth, and bony arch expansion efficiency between the three groups were measured and compared.

**Results:**

The success rates of arch expansion in the unfused, initial fusion, and complete fusion groups were 100%, 100%, and 83.3%, respectively. Expansion of the MBT in the first molar position was 4.11 ± 1.10 mm, 4.07 ± 0.42 mm, and 2.18 ± 0.66 mm in the unfused, initial fusion, and complete fusion groups, respectively (*P* < 0.05). The corresponding bony arch expansion efficiency was 58.82 ± 6.56%, 54.58 ± 8.65%, and 37.88 ± 4.36%, respectively, without statistical difference (*P* > 0.05).

**Conclusion:**

Patients with different degrees of MBT confluence and treated with MARPE had significant differences. Observation of the degree of MBT fusion before arch expansion predicts MARPE efficacy.

## Introduction

Transverse maxillary deficiency is a common type of malocclusion that may damage the facial esthetics of patients. The transverse maxillary deficiency may also lead to a series of problems, including mandibular retrusion, facial asymmetry, temporomandibular disorders syndrome, and oral breathing, due to cranio-maxillofacial structure complexity. The options for the treatment plan vary based on different physiologic ages and maturation of the midpalatal sutures. It has been reported that the tooth-tissue-supported expander (Hass) or tooth-supported expander (Hyrax) has significant efficacy in the treatment of patients at the early or peak stage of growth and development [1]. Adult patients with maxillary narrow dental arch require surgical-assisted maxillary expansion to open the fused bony suture [2]. However, the tooth-supported expander may leave irreversible side effects (buccal inclination of the molars, bony fenestration, gingival recession, and relapse) on the teeth among older patients. The operative treatment for adults has a high operative risk and high cost due to hospitalization treatment, so operative treatment is often declined by patients [3].

To address the aforementioned series of problems, micro-implant-assisted rapid maxillary palatal expansion (MARPE) was adopted clinically in 2010 [4] with the mini-screw implant serving as the palatal anchorage in MARPE treatment. The tooth movement side effect is reduced, and the effect of bone expansion is increased due to the direct effect on the basal bone caused by the orthopedic force of the expander. Studies showed multiple factors affecting the result of arch expansion, such as physiologic age, maturation of the midpalatal sutures, bone age, and midpalatal suture density ratio [5–7]. Different levels of midpalatal suture maturation were observed in the group with the same histologic age, although age is a fundamental factor affecting the efficacy of MARPE [5, 8]. Studies have shown that the bony effect generated by MARPE is varied due to multiple factors, such as midpalatal maturation and the bony sutures around the jawbone [9, 10]. Qiu et al. [11] reported that the efficiency of bone expansion is influenced by the radiographic stages of midpalatal suture maturation [11]. However, few studies have focused on the MARPE determination effect on the effectiveness of bony arch expansion.

Therefore, the current study determined the effect of the degree of midpalatal suture fusion on MARPE efficacy based on an analysis of the difference in the upper jaw, alveolar bone, and teeth and the effectiveness of bony arch expansion of patients undergoing MARPE before and after treatment.

## Materials and methods

### Participants

This was a retrospective study approved by the Ethics Committee of Hengshui People’s Hospital (Grant NO. 2021-3-011). The informed consent forms were obtained from all patients and their parents. The patients who were admitted to the Department of Orthodontics (Hengshui People’s Hospital) for transverse maxillary deficiency and treated with MARPE between January 2022 and August 2024 were recruited as the subjects for the analysis.

The inclusion criteria were as follows: ① between 10 and 35 years of age; ② maxillary basal bone width-mandibular basal bone width < 5 mm shown on cone beam computed tomography (CBCT) and midpalatal sutures maturation between B and E stages; ③ oral clinical manifestations included bilateral or unilateral posterior crossbite combined with mandible deviation, narrowing of upper and lower jaws, and lingually inclined mandibular posterior without crossbite; and ④ healthy periodontium without wearing oral expander appliances before MARPE treatment.

The exclusion criteria were as follows: ① craniofacial congenital deformity, such as cleft lip and palate; ② dental posterior crossbite; ③ bone metabolic disorders or drug administration affecting bone metabolism; and ④ other systemic diseases, such as obesity.

### MARPE treatment

**Figure 1:**
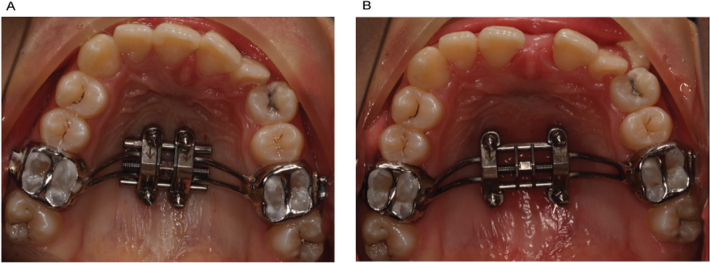
(A) Before MARPE; (B) After MARPE.

Maxillary skeletal expansion (MSE) (Shinye, Jinhua, China; Figure 1A, 1B) was used for each patient. The expander, the position of the mini-screw implant, and the MARPE treatment were confirmed by the same physician. The personalized molar band retention was designed on the first molar, and the position of the expander was placed posteriorly rather than anteriorly when the bone density at the top palatine was sufficient. Glass ionomer cement (GIC) (3M Deutschland GmbH, Germany) was used to connect the expander to the first molar of the upper jaw, and four anchorage micro-implants (self-drilling [diameter, 1.8 mm; length, 12 mm]; PT Tac, PROTECT, Huzhou, China) were implanted under local anesthesia of the pre-set positioning hole after 30 min and hitched through the nasal floor and maxillary double-layer cortical bone. The expander was rotated twice daily (morning and evening) for 0.13 mm each time from the second day after installation. The expansion effectiveness and gap appeared between the incisors were recorded to provide a daily record of the expansion. The expansion effectiveness and the gap appeared between the incisors were recorded. The patients were informed of any reduction in effectiveness or suspension of arch expansion if discomfort persisted. The patients were informed of changing the rotation effectiveness from once every 2 days or twice every 3 days (i.e. low speed expansion) to reduce tissue damage. The expansion was suspended when the upper palatal cusp was in the position of the lower palatal cusp, which was a common clinical indication for stopping the expansion of the arch. Imaging indications: Apical Palatal Width Scores (APWs)-CT index: 5 mm, Basal Arch Width Score (BAWs)-CT index: −0.39 + 1.87 mm. The nasal ventilation condition has improved. This is an indication that the drawing of the bow has ended. If the arch has been expanded for 8 to 9 days and there is no gap in the palatal slit on the X-ray, it indicates that the arch has not been expanded, and the expansion has failed.

For patients with narrow dental arches, after the arch expansion is completed, the arch expander is fixed with photocurable resin or 0.025 mm ligation wire. After maintaining for 3 months, the second-stage orthodontic treatment will be carried out, during which no clinical treatment will be performed. The MARPE device will be removed 9 months later. For patients with maxillary dysplasia accompanied by maxillary dysplasia, after the expansion of the arch is completed, the arch should be retracted for 1 week, twice a day, and 1/4 turn each time, expand the bow for another week, twice a day, and 1/4 turn each time, then stop the expansion and contraction for 2 weeks, and perform forward traction for 4 weeks. Three months later, the bow was retracted and expanded for 1 week each and then stopped for 2 weeks. The purpose of this clinical treatment is to release the bone sutures in order to promote more maxillary migration. The MARPE device will be removed 9 months later for these patients.

### CBCT data acquisition and head positioning

#### Data acquisition and head location

The CBCT scan (KaVo Dental GmbH, Biberach, Country) was performed before arch expansion (T0) and the same day after the arch expansion was completed (T1). The position of the patients was adjusted allowing the face ministry midline sagittal cut-side and frankfort plane to be parallel to the ground plane. The scanning area was the superior orbital rim-chin. The relevant parameters were adjusted. The voltage was 120 kV, the current was 36.12 mA, and 360° rotation scanning was performed. The resolution and exposure time were 0.250 mm and 40 sec, respectively. The image was saved in the ‘dicom’ format. The data were imported into InVivo software (Anatomage, San Jose, CA, USA) for the items related to head calibration, positioning, and measurement, which were operated by the same physicians. The relevant data were measured three times, and the mean value was calculated. A coefficient test was performed with the correlation coefficient > 0.9.

#### Head positioning

The CBCT image was imported into the Invivo 5 software for head position calibration. The cross-section was parallel to the plane created by the anterior nasal spine (ANS) point, posterior nasal spine (PNS) point, and the lowest point of the left and right infraorbital rims. The midline sagittal cut-side passed through the nasion and the midpoint of the anterior rim of the foramen magnum. The horizontal baseline of the coronary plane was parallel to the connecting line of the infraorbital rim, and the vertical baseline was superimposed with the facial midline.

### Acquisition of the datum plane and the criteria for the group division

#### Acquisition of the measured plane

Palatal plane: The level of the midpalatal suture expansion was clearly demonstrated according to the ANS and PNS points.

The coronary plane of the upper first molar: The coronary plane passing through the bilateral apical point of the maxillary first molars palatal root was selected.

The coronary plane of the upper first premolar: The clip plane of the image demonstrating the root canals in the apical part of the bilateral maxillary first premolars was selected.

### Group division

The morphology of the midpalatal suture in the palatal plane of the patients on the CBCT was observed, and the staging method for palatal sutures proposed by Angelieri et al. [12] was used for comprehensive evaluation. A total of 46 patients were screened. Four patients had loose implants, and two had incomplete original data, who were excluded from the screened patients. Finally, a total of 40 patients were included and divided into three groups, including 18 in the unfused group (B and C stages), 10 in the initial fusion group (D stage), and 12 in the complete fusion group (E stage). The morphology of the midpalatal suture in the unfused group was the curved high-density stitching or the two stitching lines close and parallel to each other. The trabecular bone imaging existed between the two stitching lines. The morphology of the midpalatal sutures in the initial fusion group gradually matured from the posterior-to-anterior midpalatal sutures, while the morphology of the midpalatal sutures in the complete fusion group had completely disappeared stitching with closure of the midpalatal sutures.

### Measurement items

**Figure 2:**
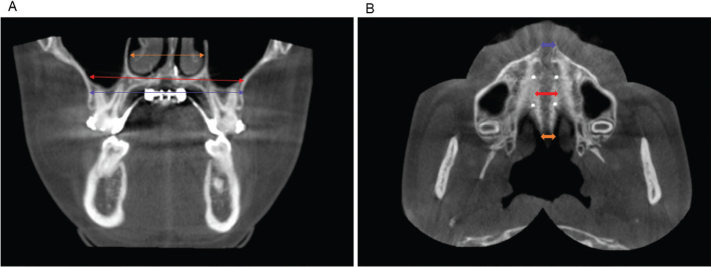
Measurement items for the jawbone. (A) The orange arrow shows nasal width, the red arrow shows maxillary base bone width, and the blue arrow shows maxillary alveolar bone width. (B) The blue arrow shows the opening distance of the midpalatal sutures at the maxillary first molar at the ANS point, the red arrow shows the opening distance of the midpalatal sutures at the M1, and the orange arrow shows the opening distance of the midpalatal sutures at the PNS point.

Select the section that can display the root canal images of the apical area of the palatal root of the bilateral first permanent molars in the maxilla. The coronal surface of the maxillary first molars passes through the center of the apical tips of the palatal roots of the bilateral maxillary first permanent molars. If the root tips of the palatal roots of the maxillary first molars are not in the same coronal plane, the central coronal plane should be selected. The measurement items for the jawbone are based on the above selected plane, as shown in Figure 2.

(1) Width of the nasal cavity: The distance between the outermost point of the inner surface of the nasal cavity in the coronal plane where the bilateral maxillary first molars were located.
(2) Width of the maxillary base bone: The distance of the lines between the points of the maxillary base bone on both sides parallel to the base line of the nasal cavity.
(3) Width of the midpalatal sutures at the maxillary first molar: The width of the middle cleft of the upper palate in the axial cross-section parallel to the base surface of the nasal cavity on the coronal plane where the apical point of the palatal root of the first maxillary molar was located.
(4) Width of the midpalatal sutures of the ANS: The distance of the midpalatal sutures between the points of the bilateral ANSs.
(5) Width of the midpalatal sutures of the PNS: The distance of the midpalatal sutures between the points of the bilateral PNSs.

**Figure 3:**
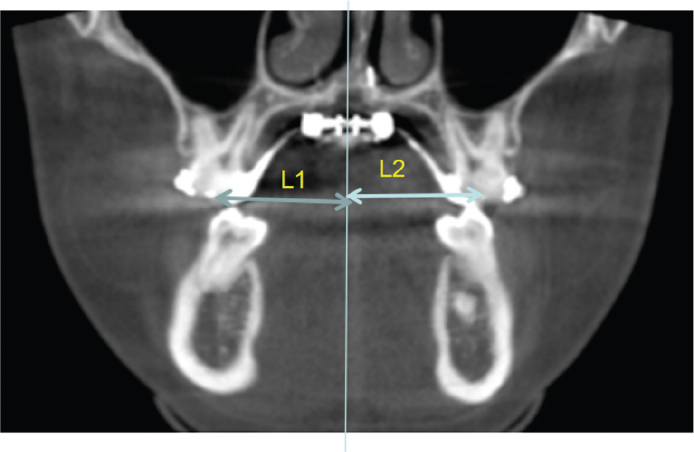
Width between the first molars. L1 is the distance from the crown of the first molar of the right upper jaw to the midline; L2 is the distance from the crown of the first molar of the left upper jaw to the midline.

**Figure 4:**
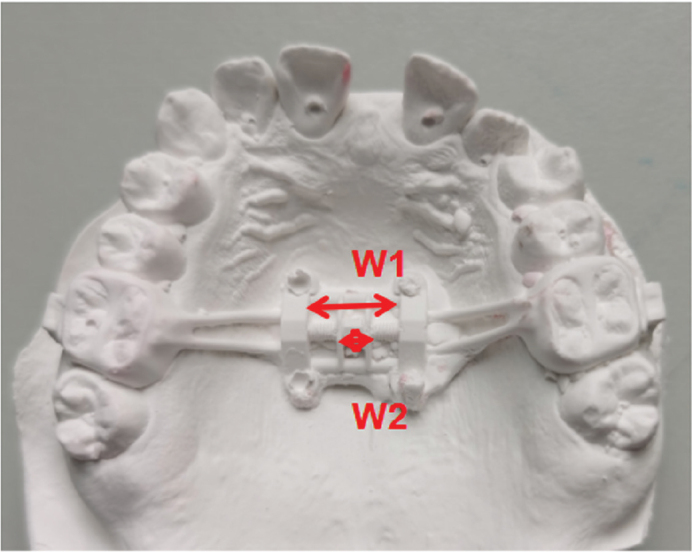
The opening amount of the expansion of the dental cast. W1: Width of expander after MARPE; W2: Width of expander before MARPE.

The measurement items for the alveolar bone are shown in Figures 3 and 4.

(1) Width of the maxillary alveolar bone: The distance between the points of the maxillary left and right alveolar bone parallel to the base line.
(2) Height of the alveolar spine of the maxillary first molar on the left and right sides: The vertical distance between the alveolar spine at the lowest point of the mesial buccal root and the base line.

### The measurement items for the teeth changes (Figure 5)

(1) Width between the first molars: The sum of the distances between the points of the central fovea and the central sagittal plane on the left and right sides.
(2) The inclination of the first molar on the left and right sides: The included angle between the connecting line for the root canal orifice of the maxillary first molar palatal root and the base line.
(3) The inclination of the first premolar on the left and right sides: The included angle between the connecting line for the central fovea of the maxillary first premolar palatal root tip and the base line.
(4) Teeth-induced palatal expansion: The changes in the width between the first molars and the changes in the width of the alveolar bone at the maxillary first molar.

**Figure 5:**
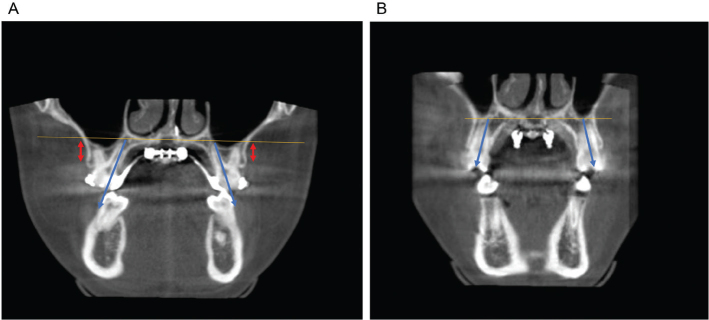
Measurement items for alveolar bone and inclination of the teeth. (A) The red arrow shows the alveolar ridge height at the left side and at the right side, the angle in blue arrows shows the molar inclination at both sides. (B) Blue arrows show molar inclination at both sides.

### The thickness of the midpalatal suture in the initial fusion group and the complete fusion group

In the sagittal view, the image was rotated so that the midpoint of the posterior margin of the incisor and the PNS point were on the same sagittal line. Starting from the midpoint of the posterior margin of the incisor and extend backward to the PNS point, the thickness of the midpalatal suture (MBT) was measured at an interval of 4 mm. The measurement points were marked in sequence as MBT4, MBT8, MBT12, MBTl6, MBT20, MBT24, MBT28, MBT32, and MBT36. Then, vertical lines from each measurement point were drawn. The overlapping part of the vertical lines with the hard palate was the MBT at that point.

### Effectiveness of the bony palatal expansion

(1) The amount of opening for the expander appliance: The D-value of the expander width before and after palatal expansion.
(2) The amount of opening for the MBTs: The width of the MBTs at the maxillary first molar.
(3) The effectiveness of the bony palatal expansion: The amount of opening for the MBTs and the amount of the opening for the expander appliance.

### Success of arch expansion

The clinical criterion for successful arch expansion was the anterior and posterior opening of the palatal sutures. It was also very important to measure the front and back parts. In this study, the opening amounts of the ANS, PNS, and the palatine cleft at the maxillary first molar were measured, and it was further determined that the palatine cleft opens in a triangular pattern, with the anterior part being the widest.

### Statistical methods

The data were processed using the SPSS 26.0 software. Descriptive statistics were performed using continuous variables, which are expressed by the mean value and standard deviation. The categorical variables are expressed as percentages. The paired t-test or Wilcoxon’s rank sum test was used to evaluate the changes in the treatment based on the normality of the data. To compare the inter-group difference, a one-way analysis of variance (ANOVA) was used, and the Least Significant Difference (LSD) test was used for analysis of the inter-group difference. A chi-square test was used to compare the inter-group occurrence effectiveness distribution, and a *P* < 0.05 was considered statistically different.

## Results

A total of 40 patients (14 males and 26 females) between 10 and 35 years of age (mean, 16.72 ± 2.81 years) were included and divided into three groups: 18 in the unfused group (6 males and 12 females); 10 in the initial fusion group (5 males and 5 females); and 12 in the complete fusion group (4 males and 8 females). See Table 1 for the baseline data of the included patients.

**Table 1:** Baseline data for the included patients with different levels of midpalatal maturation.

Indicators	Total value	Unfused group	Initial fusion group	Complete fusion group	*P*
Gender					< 0.001
Male	15	6	5	4	
Female	25	12	5	8	
Average age (year)	18.3 ± 6.9 (10.0–35.0)	12.1 ± 1.5 (10.0–14.6)	18.7 ± 1.8 (15.5–21.3)	27.3 ± 3.2 (24.0–35.0)	< 0.001
Time for palatal expansion	19.3 ± 6.2	13.6 ± 1.0	19.7 ± 1.1	27.6 ± 2.3	< 0.001
Success rate					< 0.001
Success	38	18	10	10	
Failure	2	0	0	2	
Rate of success (%)	95	100	100	83.3	

### Time of the initiative palatal expansion and rate of success for the MBT expansion

The total rate of success for the palatal expansion among all patients was 95%. The rate of success for the palatal expansion among the unfused, initial fusion, and complete fusion groups was 100%, 100%, and 83.3%, respectively. Two patients had midpalatal expansion failure in the initial fusion group (Table 1).

### Comparison of bony palatal expansion effectiveness

There were significant differences in palatal expansion effectiveness among the unfused, initial fusion, and complete fusion groups (*P* < 0.05), while there was no statistical difference in the palatal expansion effectiveness between the unfused and initial fusion groups (*P* > 0.05). However, there was a significant difference in the palatal expansion effectiveness between the unfused and complete fusion and between the initial fusion and complete fusion groups, respectively (*P* < 0.05). The opening amount for the expander appliance among the unfused, initial fusion, and complete fusion groups was significantly different (*P* < 0.05). No statistical difference was detected in the opening amount for the expander appliance between the unfused and initial fusion groups (*P* > 0.05), while the opening amount for the expander appliance was significantly different between the unfused and complete fusion and between the initial fusion and complete fusion groups, respectively (*P* < 0.05). There was a significant difference in the opening amount for the MBTs among the three groups (*P* < 0.05). No statistical difference existed in opening amount for the MBTs between the unfused and initial fusion groups (*P* > 0.05). There was a significant difference in the opening amount for the MBTs between the unfused and complete fusion and between the initial fusion and complete fusion groups, respectively (*P* < 0.05). To summarize, as the MBT maturation increased, the palatal expansion effectiveness and opening amount for MBTs decreased (Table 2).

**Table 2:** Comparison of bony palatal expansion for patients with different levels of midpalatal maturation in the three groups after MARPE treatment.

Indicators	Unfused group (18 cases)	Initial fusion group (10 cases)	Complete fusion group (12 cases)	*F*	*P*
Opening amount for the expander appliance	6.99 ± 0.56	7.14 ± 1.23	5.64 ± 0.75	10.347	< 0.001
Opening amount for the midpalatal sutures	4.11 ± 1.10	4.07 ± 0.42	2.18 ± 0.66	26.26	< 0.001
Bony palatal expansion effectiveness	58.82 ± 6.56	54.58 ± 8.65	37.88 ± 4.36	37.686	< 0.001

The fusion conditions of the midpalatal sutures in the initial fusion group and the complete fusion group were shown in Table 3. There was a statistically significant difference in MBT at 4 mm (*P* = 0.036) and 24 mm (*P* = 0.032) behind the incisor holes between the two groups (Table 4). The MBT at 24 mm behind the incisor was negatively correlated with the maxillary arch expansion rate (MER) (*r* = −0.764, *p* = −0.000, *P* < 0.001, Table 5).

**Table 3:** The fusion conditions of the midpalatal sutures in the initial fusion group and the complete fusion group.

Indicators	Initial fusion group (10 cases)	Complete fusion group (12 cases)
The entire midpalatal suture fusion	0	12
Horizontal plate fusion of the palatine bone	10	12
Anterior maxillary fusion	0	12

**Table 4:** The thickness of the midpalatal suture in the initial fusion group and the complete fusion group.

The thickness of the midpalatal suture	Initial fusion group (10 cases)	Complete fusion group (12 cases)	*t*	*P*
	Mean value ± standard deviation	Mean value ± standard deviation		
MBT4	4.38 ± 1.24	5.61 ± 1.31	−2.247	0.036
MBT8	6.96 ± 1.26	6.29 ± 1.74	1.018	0.321
MBT12	5.47 ± 1.31	5.64 ± 1.09	−0.316	0.755
MBT16	5.46 ± 1.38	6.67 ± 1.76	−1.766	0.093
MBT20	6.99 ± 0.91	7.26 ± 0.83	−0.731	0.473
MBT24	6.98 ± 0.88	7.93 ± 1.02	−2.308	0.032
MBT28	7.78 ± 0.88	7.80 ± 1.18	−0.034	0.973
MBT32	7.25 ± 0.73	6.56 ± 1.32	1.478	0.155
MBT36	5.80 ± 1.08	5.89 ± 0.94	−0.221	0.827

**Table 5:** The correlation analysis between the thickness of the midpalatal suture palatal expansion effectiveness rate.

Measurements	Palatal expansion effectiveness	
	*R*	*P*
Age	−0.630	0.002
Grouping	−0.796	0.000
MBT4	−0.405	0.062
MBT8	0.068	0.763
MBT12	−0.346	0.115
MBT16	−0.353	0.107
MBT20	−0.045	0.844
MBT24	−0.764	< 0.001
MBT28	0.114	0.614
MBT32	0.307	0.164
MBT36	0.149	0.508

The changes in jawbone, alveolar bone, and teeth among the patients in three groups after the MARPE treatment are shown in Supplemental file.

## Discussion

In the current study, the success rates of arch expansion in the unfused, initial fusion, and complete fusion group were 100%, 100%, and 83.3%, respectively. While no significant difference was detected in the bony arch expansion efficiency between the non-fusion and initial fusion groups, significant differences were found between the non-fusion, the initial fusion, and complete fusion groups. These results showed that the patients with different degrees of middle palatal suture confluence had differences in MARPE efficiency, indicating that an increase in the midpalatal maturation will lead to greater maxillary resistant forces, and the palatal expansion effectiveness will decrease.

According to Angelieri et al. [12], the C stage in the maturation of the midpalatal sutures refers to the critical stage of traditional transverse maxillary deficiency, and for patients in the D and E stages, the mini-screw implant and the maxillary palatal expansion may be combined for the treatment. The patients were classified according to Angelieri et al. [12] and divided into three groups based on CBCT midpalatal suture maturation before expansion: unfused group (B and C stages); initial fusion group (D stage); and complete fusion group (E stage). The patients with complete fusion were included in the study, and a retrospective study involving midpalatal sutures from the unfused, initial fusion, and complete fusion stages was conducted to provide literature support on choosing the methods of palatal expansion for patients at different midpalatal suture maturation stages with transverse maxillary deficiency.

According to Salmoria et al. [13], the MARPE treatment failed in 6 of the E-stage patients at midpalatal suture maturation with the failure rate higher than that in the current study (5%). The basis for this finding may be related to individual differences, such as the bone density at the midpalatal sutures and the different implanting position of the palatal implant anchorage. This study showed the fusion conditions of the midpalatal sutures in the initial fusion group and the complete fusion group in Table 3. In the initial fusion group, all 10 cases showed horizontal plate fusion of the palatine bone, but no anterior maxillary fusion, and no entire midpalatal suture fusion, while the complete fusion group showed all fusion in anterior maxillary fusion and horizontal plate fusion of the palatine bone. From previous literature [12, 13] and the clinical images of this study, it can be observed that in the initial fusion group (D) stage, the palatine cleft of the palatine bone segment has completed fusion, and the fusion process has advanced from back to front. At this stage, the midpalatal suture within the palatine bone is not visible. Though the midpalatal suture within the maxillary segment had not yet fused, two high-density line shadows separated by a narrow low-density gap can still be seen. In the complete fusion group (E) stage, the midpalatal suture within the maxilla segment has completed fusion. This midpalatal suture might be invisible in some part of the maxilla, but its bone density was consistent with that of other areas of the palate. In the complete fusion group, the entire middle palatal suture has fused, and the resistance to arch expansion has increased. It is speculated that this is the reason for the failure of MARPE therapy. In addition, the MBT at 24 mm behind the incisor was negatively correlated with the MER (Table 5). It is speculated that a larger MBT may lead to greater resistance when the midpalatal suture was dilated, which, in turn, will increase the difficulty of dilating the midpalatal suture and ultimately result in the failure of MARPE treatment.

Age was used as the criterion for group division by Jia [5], and the ratio of palatine suture expansion at the first molar to increase in spiral width of arch expansion was 69.4%, 51.3%, 39.0%, and 29.8% in the different age groups after the MARPE treatment. Twenty-two patients treated by MARPE showed the effectiveness of maxillary bony palatal expansion (59.23 ± 17.75%) [14]. Thirty-eight patients were treated by MARPE, and the effectiveness of the maxillary bony palatal expansion was 63.0 ± 16.3% [15]. The effectiveness of the bony palatal expansion was 74% in a study conducted by Li et al. [16]. The results of the preceding studies differed from the results of the current study [5, 14–16]. The basis for the differences may reflect individual differences and the level of midpalatal suture maturation among different patients. The results of MARPE treatment also vary, which will result in statistical differences in the effectiveness of palatal expansion.

The current study results suggested that the movement of the anchorage teeth increased as midpalatal suture maturation increased, which is consistent with the findings in the Jesus et al.’s study [17]. With the increase in midpalatal suture maturation, the maxillary resistant forces further increase. Movement of the anchorage teeth is easily caused by the same forces, and the bone will be more difficult to move. Naveda et al. [18] concluded that buccal movement of the teeth increases the distance between the cemento-enamel junction and the buccal alveolar ridge spine, resulting in gingival recession and allergy of cementum. The anchorage appliance was the maxillary first molar and micro-implant in the current study, but there may be a possibility of decreased alveolar height. The maxillary mesiobuccal root protrudes significantly. The initially covered bone plate is quite thin and easily causes dehiscence of the alveolar bone in the mesiobuccal area. Therefore, we evaluated the height of the alveolar bone in the mesiobuccal area of the first molar root.

The height of the alveolar bone of the maxillary first molar in the patients from the three groups after the treatment of palatal expansion was shown to decrease by 0.28–1.94 mm, which was consistent with the study conducted by Lim et al. [19]. The results of which indicated that the height of the alveolar bone of the maxillary first molar in adult patients decreased by 0.74 mm after MARPE treatment and lower than a tooth-borne rapid palatal expander (RPE), while the alveolar bone of the anchorage first molar in the mesiobuccal root area decreased by 3.8 mm after treatment with a tooth-borne RPE during puberty [19]. No statistical difference in alveolar bone loss was detected in the three groups of patients, suggesting that MARPE would generate more bone effect compared to traditional RPE treatment. The decrease in the measured height of the alveolar bone of the maxillary first molar mainly resulted from the maxillary fan-shape separation and the teeth buccal movement caused by anchorage loss. Notably, the actual alveolar bone loss of the anchorage teeth was less than the measured value. The decreased alveolar of the first molar height in adult patients may be due to the thin buccal bone plate and anchorage loss.

The amount of transversal expansion for midpalatal sutures gradually decreases from anterior-to-posterior when observed at the palatal plane level. The mean value of the width of the midpalatal sutures among unfused, initial fusion, and complete fusion groups increased by 4.77, 4.42, and 2.52 mm at the ANS position and increased by 3.43, 2.95, and 1.73 mm at the PNS position, respectively. Taken together, these results suggested that the midpalatal suture was expanded in a triangle-like shape with the widest expansion in the anterior region. This difference may be caused by resistance of the sphenoidal pterygoid plate, the position of MARPE expander, which is located at the anterior center of the maxillary resistance, and the combined movement of the zygomaticomaxillary complex (ZMC) [20].

The evaluation of the midpalatal suture maturation stages through CBCT before treatment has a positive guiding role in predicting the rate of MARPE treatment success. Different types of anchorage implant should be chosen based on the observation of the thickness and density of the palatal bone tissue [21, 22]. The appropriate type of expander should be chosen based on the midpalatal suture maturation stages, prediction of the bony palatal expansion ratio, and calculation of the bony palatal expansion. The patients should be provided with multiple treatment plans combined with midpalatal suture stages. For example, the surgical-assisted maxillary palatal expansion should be suggested to patients with high midpalatal suture maturation to reduce the dental and periodontal tissue injury of the patients and the palatal expansion failure rate [9, 23].

This study was limited by the small sample size from a single center. Also, the voxel size of the CBCT images (regarding the spatial resolution) may be limited for 100% accurate measurement of the midpalatal suture opening, which may lead to some bias to the results in some degrees. Therefore, the results need further validation in a multi-center study with a large sample size, considering using other imaging tools.

In conclusion, an increase in midpalatal maturation may lead to greater maxillary resistant forces, and the palatal expansion effectiveness may decrease.

## Supplementary Material



## Data Availability

The data are available from the corresponding author, SL, upon reasonable request.
