# Supplementary material for: Relationship between the efficacy of micro-implant-assisted rapid maxillary arch expansion and maturation of the midpalatal sutures
Source: Acta Odontol Scand. 2026 May 4;85:45902. doi: 10.2340/aos.v85.45902 (PMC13151780; doi:10.2340/aos.v85.45902)
Supplement: Supplementary file 1 [file AOS-85-45902-s1.pdf]

Supplementary material has been published as submitted. It has not been copyedited or typeset by Acta Odontologica Scandinavica.

Supplemental file. Changes in the jawbone, alveolar bone, and teeth among the patients in the three groups after MARPE treatment

A significant increase was demonstrated in the maxillary width at the base bone, alveolar bone, and teeth level for the patients in all groups after palatal expansion ($P < 0.05$). The differences in the maxillary palatal expansion changes in the groups are shown in Supplemental Table 4 as below. The opening amount for the mid-palatal sutures at the first molars among the patients in the three groups were 4.11±1.10 mm, 4.07±0.42 mm, and 2.18±0.66 mm, respectively. The corresponding bony palatal expansion effectiveness was 58.82 ±6.56%, 54.58±8.65%, and 37.88±4.36%. The amount expansion for the mid-palatal sutures and the effectiveness of the bony palatal expansion decreased as mid-palatal suture maturation increased, yet no significant statistical difference was found on the indicators for expansion amount of the mid-palatal sutures and the effectiveness of the bony palatal expansion between the unfused and initial fusion groups ($P > 0.05$). The expansion amount of the mid-palatal sutures and the effectiveness of bony palatal expansion was significantly different in the complete fusion group compared to the unfused and initial fusion groups ($P < 0.05$), indicating the greater maxillary resistant forces and the increased difficulty in expansion due to the complete fusion of the mid-palatal sutures in the complete fusion group. The amount of transversal expansion for the mid-palatal sutures gradually decreased from anterior-to-posterior when observed at the palatal plane level. The mean value of the width of the mid-palatal sutures among different groups increased by 4.77 mm, 4.42 mm, and 2.52 mm at the ANS position, respectively and increased by 3.43 mm, 2.95 mm, and 1.73mm at the PNS position, respectively. Taken together, these results suggested that the mid-palatal suture expanded in a triangle-like shape with the widest expansion in the anterior region. No statistical difference in alveolar bone height changes for the first molar was detected among the patients in the three groups after expansion ($P > 0.05$). The width between the crowns of the maxillary first molars increased by 5.53 mm, 5.88 mm, and 5.00 mm in the unfused, initial fusion, and complete fusion groups, respectively and no statistically significant difference was detected among the three groups ($P > 0.05$). The buccal inclination of the maxillary first molar increased significantly after expansion ($P < 0.05$). In addition to the inclination of the left-sided molar, the changes in the buccal inclination of the first molar'right side and the first premolar for the patients among the three groups were not significantly different ($P > 0.05$;

Supplemental Tables 1-4).

Supplemental Table 1: Comparison of changes in measurement items related to the maxillary bone, alveolar bone, and teeth for the patients in the unfused group before and after palatal expansion（n=18）

Items	Before expansion		After expansion		Changes after expansion		t	*p*
	Mean Value	Standard Deviation	Mean Value	Standard Deviation	Mean Value	Standard Deviation		
Jawbone changes								
Width of the nasal cavity	30.33	1.00	32.62	0.99	2.29	0.52	18.65	0.000
Width of the maxillary base bone	61.89	3.11	65.88	3.10	3.96	0.39	42.68	0.000
Width of the mid-palatal suture mid-point	0	0	4.07	0.42	4.07	0.42	41.02	0.000
Width of the mid-palatal sutures at the anterior nasal spine	0	0	4.77	0.55	4.77	0.55	37.03	0.000
Width of the mid-palatal sutures at the posterior nasal spine	0	0	3.43	0.35	3.43	0.35	41.305	0.000
Alveolar bone changes								
Width of the maxillary alveolar bone	61.20	2.92	65.18	2.87	3.98	0.30	55.30	0.000
Height of the alveolar spine on the right side of the first molar	13.80	2.38	13.46	2.26	-0.34	0.77	0.44	0.663
Height of the left alveolar ridge on the first molar	13.45	1.44	12.81	1.77	-0.64	1.30	-2.09	0.052
Teeth changes								
Width between the first molars	47.27	2.38	52.80	2.34	5.53	1.27	18.48	0.000
Inclination of the molars on the right side	109.57	4.88	113.57	4.91	4.00	0.57	29.78	0.000
Inclination of the molars at the right side	109.86	7.91	112.93	7.71	3.07	2.76	4.72	0.000
Inclination of the premolars on the right side	92.41	4.63	92.38	4.58	-0.03	0.11	-0.99	0.336

Inclination of the premolars on the left side	95.30	8.01	95.32	7.81	0.03	0.51	0.209	0.837

Note：A $P < 0.05$ indicates that the difference has statistical significance.

Supplemental Table 2: Comparison of changes in the measurement items related to the maxillary bone, alveolar bone, and teeth for patients in the initial fusion group before and after palatal expansion（n=10）

Items	Before expansion		After expansion		Changes after expansion		t	p
	Mean Value	Standard Deviation	Mean Value	Standard Deviation	Mean Value	Standard Deviation		
Jawbone changes								
Width of the nasal cavity	30.14	1.70	32.54	1.64	2.40	0.41	18.58	0.000
Width of the maxillary base bone	62.27	4.18	65.37	4.18	3.11	0.11	85.81	0.000
Width of the mid-palatal suture mid-point	0	0	4.11	1.10	4.11	1.10	11.85	0.000
Width of the mid-palatal sutures at the anterior nasal spine	0	0	4.42	0.65	4.42	0.65	21.64	0.000
Width of the mid-palatal sutures at the posterior nasal spine	0	0	2.95	0.64	2.95	0.64	14.68	0.000
Alveolar bone changes								
Width of the maxillary alveolar bone	60.04	3.43	63.74	3.26	3.70	0.74	15.76	0.000
Height of the first molar alveolar ridge on the right side	16.64	2.67	16.14	2.85	-0.50	0.78	-2.03	0.073
Height of the first molar alveolar ridge on the left side	15.76	2.65	15.27	3.06	-0.50	0.83	-1.88	0.093

Teeth changes								
Width between the first molars	46.84	4.94	52.72	4.71	5.88	1.17	15.86	0.000
Inclination of the molar on the right side	110.01	8.40	114.17	8.31	4.16	0.36	36.86	0.000
Inclination of the molar on the left side	105.86	6.52	110.60	6.71	4.74	0.88	17.10	0.000
Inclination of the premolar on the right side	92.58	4.24	93.29	5.13	0.71	1.34	1.670	0.129
Inclination of the premolar on the left side	89.14	6.20	90.35	5.87	1.21	4.40	0.870	0.407

Supplemental Table 3: Comparison of changes in the measurement items related to the maxillary bone, alveolar bone, and teeth for the patients in the complete fusion group before and after palatal expansion（n=12）

Items	Before expansion		After expansion		Changes after expansion		t	*p*
	Mean Value	Standard Deviation	Mean Value	Standard Deviation	Mean Value	Standard Deviation		
Jawbone changes								
Width of the nasal cavity	31.28	1.89	32.96	1.86	1.68	0.16	33.410	0.000
Width of the maxillary base bone	59.88	3.35	61.76	3.31	1.88	0.18	32.859	0.000
Width of the mid-palatal suture mid-point	0	0	2.18	0.66	2.18	0.66	10.384	0.000
Width of the mid-palatal sutures at the anterior nasal spine	0	0	2.52	0.32	2.52	0.32	24.571	0.000
Width of the mid-palatal	0	0	1.73	0.45	1.73	0.45	12.083	0.000

sutures at the posterior nasal spine								
Alveolar bone changes								
Width of the maxillary alveolar bone	60.83	2.60	63.00	2.64	2.17	0.46	14.907	0.000
Height of the first molar alveolar ridge on the right side	15.11	2.09	14.71	2.13	-0.40	0.56	-2.251	0.051
Height of the first molar alveolar ridge on the left side	15.01	2.14	14.52	2.10	-0.49	0.79	-1.970	0.080
Teeth changes								
Width between the first molars	45.45	3.14	50.45	3.32	5.00	2.99	5.280	0.001
Inclination of the molar on the right side	99.74	11.61	105.33	11.23	5.60	3.36	5.270	0.003
Inclination of the molar on the left side	101.40	9.19	109.65	8.48	8.25	4.04	6.457	0.000
Inclination of the premolar on the right side	92.52	4.38	92.66	4.30	0.143	0.54	0.842	0.422
Inclination of the premolar on the left side	88.98	5.62	89.14	5.60	0.15	0.35	1.410	0.192

Supplemental Table 4: Comparison of changes in the values related to the jawbone, alveolar bone, and teeth for the patients in the three groups with different mid-palatal suture maturation before and after palatal expansion（n=40）

Items	Unfused group		Initial fusion group		Complete fusion group		F	*p*
	Mean Value	Standard Deviation	Mean Value	Standard Deviation	Mean Value	Standard Deviation		

Jawbone changes								
Width of the nasal cavity	2.29	0.52	2.40	0.41	1.68	0.16	8.78	0.001
Width of the maxillary base bone	3.96	0.39	3.11	0.11	1.88	0.18	160.40	0.000
Width of the mid-palatal sutures	4.07	0.42	4.11	1.10	2.18	0.66	26.26	0.000
Width of the mid-palatal sutures at the anterior nasal spine	4.77	0.55	4.42	0.65	2.52	0.32	60.50	0.000
Width of the mid-palatal sutures at the posterior nasal spine	3.43	0.35	2.95	0.64	1.73	0.45	42.94	0.000
Alveolar bone changes								
Width of the maxillary alveolar bone	3.98	0.30	3.70	0.74	2.17	0.46	45.24	0.000
Height of the first molar alveolar ridge on the right side	-0.34	0.77	-0.50	0.78	-0.40	0.56	0.45	0.641
Height of the first molar alveolar ridge on the left side	-0.64	1.30	-0.50	0.83	-0.49	0.79	0.09	0.914
Teeth changes								
Width between the first molars	5.53	1.27	5.88	1.17	5.00	2.99	0.59	0.562
Inclination of the molar on the right side	4.00	0.57	4.16	0.36	5.60	3.36	2.85	0.072
Inclination of the molar on the left side	3.07	2.76	4.74	0.88	8.25	4.04	18.14	0.000
Inclination of the premolar on the right side	-0.03	0.11	0.71	1.34	0.143	0.54	3.25	0.051
Inclination of the premolar on the left side	0.03	0.51	1.21	4.40	0.15	0.35	0.94	0.399

Teeth-induced palatal expansion	1.55	1.36	2.18	1.27	2.83	2.04	14.31	0.000
